# Construction of the model for predicting prognosis by key genes regulating EGFR-TKI resistance

**DOI:** 10.3389/fgene.2022.968376

**Published:** 2022-11-25

**Authors:** Jinke Zhuge, Xiuqing Wang, Jingtai Li, Tongyuan Wang, Hongkang Wang, Mingxing Yang, Wen Dong, Yong Gao

**Affiliations:** ^1^ Department of Respiratory Medicine, Hainan Cancer Hospital, Haikou, China; ^2^ Department of Breast Surgery, The First Affiliated Hospital of Hainan Medical University, Haikou, China; ^3^ Department of Clinical Laboratory, Fuyang Second People’s Hospital, Fuyang Infectious Disease Clinical College, Anhui Medical University, Fuyang, China

**Keywords:** drug resistance, EGFR-TKI, LUAD, nomogram, prognosis prediction

## Abstract

**Background:** Previous studies have suggested that patients with lung adenocarcinoma (LUAD) will significantly benefit from epidermal growth factor receptor tyrosine kinase inhibitors (EGFR-TKI). However, many LUAD patients will develop resistance to EGFR-TKI. Thus, our study aims to develop models to predict EGFR-TKI resistance and the LUAD prognosis.

**Methods:** Two Gene Expression Omnibus (GEO) datasets (GSE31625 and GSE34228) were used as the discovery datasets to find the common differentially expressed genes (DEGs) in EGFR-TKI resistant LUAD profiles. The association of these common DEGs with LUAD prognosis was investigated in The Cancer Genome Atlas (TCGA) database. Moreover, we constructed the risk score for prognosis prediction of LUAD by LASSO analysis. The performance of the risk score for predicting LUAD prognosis was calculated using an independent dataset (GSE37745). A random forest model by risk score genes was trained in the training dataset, and the diagnostic ability for distinguishing sensitive and EGFR-TKI resistant samples was validated in the internal testing dataset and external testing datasets (GSE122005, GSE80344, and GSE123066).

**Results:** From the discovery datasets, 267 common upregulated genes and 374 common downregulated genes were identified. Among these common DEGs, there were 59 genes negatively associated with prognosis, while 21 genes exhibited positive correlations with prognosis. Eight genes (ABCC2, ARL2BP, DKK1, FUT1, LRFN4, PYGL, SMNDC1, and SNAI2) were selected to construct the risk score signature. In both the discovery and independent validation datasets, LUAD patients with the higher risk score had a poorer prognosis. The nomogram based on risk score showed good performance in prognosis prediction with a C-index of 0.77. The expression levels of ABCC2, ARL2BP, DKK1, LRFN4, PYGL, SMNDC1, and SNAI2 were positively related to the resistance of EGFR-TKI. However, the expression level of FUT1 was favorably correlated with EGFR-TKI responsiveness. The RF model worked wonderfully for distinguishing sensitive and resistant EGFR-TKI samples in the internal and external testing datasets, with predictive area under the curves (AUC) of 0.973 and 0.817, respectively.

**Conclusion:** Our investigation revealed eight genes associated with EGFR-TKI resistance and provided models for EGFR-TKI resistance and prognosis prediction in LUAD patients.

## 1 Introduction

Lung cancer is one of the most common diseases since more than 2 million new cases are detected globally every year (Ren et al., 2021; Sung et al., 2021). Based on cell type, lung cancer could be split into small-cell (15%) and non-small-cell (NSCLC, 75%) (Li et al., 2021). According to histological categorization, NSCLC is often separated into lung adenocarcinoma (LUAD), lung squamous cell carcinoma (LUSC), and large cell carcinoma (Nasim et al., 2019). LUAD comprises about 50% of all lung cancer cases (Ren et al., 2022), and the majority of LUAD cases are diagnosed in the late stages of cancer (Al-Dherasi et al., 2021). Despite advances in cancer treatment, including the use of PD1 antibodies, only 15% of LUAD patients could survive more than 5 years (Ma et al., 2020).

Despite the fact that the efficiency of therapy for advanced LUAD is still unsatisfactory, the prognosis of LUAD is starting to improve due to the emergence of novel molecular targeting therapies. The effective treatment of EGFR-TKI for EGFR mutation patients is virtually a breakthrough in personalized medicine (Wang et al., 2022). EGFR-TKI has been regarded as the first-line therapy for LUAD individuals with EGFR mutations. According to the findings of a meta-analysis, first-line EGFR-TKI substantially increased progression-free survival (PFS) when compared with chemotherapy (Lee et al., 2017). Most LUAD patients treated with EGFR-TKI will develop disease progression and resistance within a year (Wu et al., 2018). The mechanisms of EGFR-TKI resistance have not been fully investigated, and a lack of resistance-related biomarkers exists. As a result, novel indicators and models for predicting EGFR-TKI resistance are urgently required.

Here, we acquired EGFR-TKI resistance and sensitive data from online databases, and multiple datasets were analyzed to identify common genes related to EGFR-TKI resistance. Moreover, we constructed the risk score for prognosis prediction of LUAD by LASSO and Cox analysis. The associations of the risk score with clinical features and tumor microenvironment (TME) compositions were investigated. The capabilities of the risk score for the prediction of EGFR-TKI resistance and prognosis were validated in the independent datasets. Our study provides possible targets for EGFR-TKI resistance as well as models for predicting EGFR-TKI resistance and LUAD prognosis.

## 2 Materials and methods

### 2.1 Data acquisition

GEO, one of the largest public gene expression data resources, contains the gene expression data of resistant and sensitive cells to EGFR-TKI, such as gefitinib, erlotinib, and afatinib. We searched the potential datasets on GEO by keywords (gefitinib, erlotinib, afatinib, and epidermal growth factor receptor tyrosine kinase). The potential datasets were then filtered by the following requirements: 1) the expression data should come from human NSCLC cells or samples; 2) the dataset should contain at least 3 sensitive and 3 resistant NSCLC cells/samples without genetic manipulation such as knockdown of a specific gene; 3) the mRNA expression matrix should be available on the GEO platform. Among the 34 available datasets from search results for “gefitinib”, GSE34228 and GSE123066 were selected by the criteria. Among the 39 available datasets from search results for “erlotinib”, GSE80344 was selected by the criteria. Among the 8 available datasets from search results for “afatinib”, none were selected. Among the 4 available datasets from search results for “epidermal growth factor receptor tyrosine kinase”, GSE122005 and GSE31625 were selected by the criteria. The searching and filtering results were provided in Supplementary Table S1.

Among these five datasets, the discovery datasets were GSE31625 (28 erlotinib-resistant and 18 erlotinib-sensitive samples) (Balko et al., 2006) and GSE34228 (26 gefitinib-resistant and 26 gefitinib-sensitive samples) (Nakata et al., 2015). The discovery datasets were used for identifying resistance-related genes and constructing the model for predicting the resistance of a sample by expression data. Three independent datasets (GSE122005, GSE80344, and GSE123066) were used to validate the diagnosis abilities of selected genes and the model for predicting the resistance. GSE122005 contains 3 gefitinib-sensitive and 3 gefitinib-resistant lung cancer cell samples (Wu et al., 2019). GSE80344 contains 4 erlotinib-sensitive and 12 erlotinib-resistant lung cancer cell lines (Fustaino et al., 2017). GSE123066 contains 3 gefitinib-sensitive samples and 3 gefitinib-resistant cells.

In order to construct the risk score model for predicting the prognosis of LUAD patients, RNA-seq expression values (level 3, raw count) and clinical records were retrieved from the TCGA-LUAD database by the TCGAbiolinks package (Colaprico et al., 2016). An independent dataset (GSE37745) was randomly selected as the testing dataset for validating the risk score model. GSE37745 contained 226 lung cancer samples with expression values and clinical records (Botling et al., 2013).

### 2.2 Differentially expressed genes

The expression data of discovery datasets (GSE31625 and GSE34228) was downloaded by “GSE31625_series_matrix.txt.gz” and “GSE34228_series_matrix.txt.gz” from the package of GEOquery (Davis et al., 2007). Then, the expression data from these two datasets was annotated with ‘GPL96. soft’ and ‘GPL4133. soft’, respectively. The gene expression value was retained by the probe with the highest expression. DEGs were determined by analyzing the sensitive and resistant gene expression patterns using the edgeR (Robinson et al., 2010). The cut-off values for DEGs were set to *p*-values<0.05 and |log2FC|>0.5. DEGs with log2FC>0.5 were defined as upregulated DEGs, and genes with log2FC<-0.5 were defined as downregulated DEGs in resistant profiles. The important DEGs were shown as a heatmap and a volcano plot, respectively. The shared DEGs in these two discovery datasets were obtained by Wayne analysis.

### 2.3 Functional enrichment analyses

Gene ontology (GO) analysis is a common technique for studying the biological function of genetic data. It usually contains biological process (BP), cellular component (CC), and molecular function (MF). KEGG and Hallmark include a large number of well-defined pathways and their correspondent genes. To obtain functional annotations, we subsequently uploaded the common upregulated and downregulated DEGs (dDEGs) to Enrichr (https://maayanlab.cloud/Enrichr/) to process the GO, KEGG, and Hallmark signal pathway analysis (Kuleshov et al., 2016). As a result, *p*-value< 0.05 was considered statistically significant.

### 2.4 Construction of the signature

Based on the survival data and expression profiles from the TCGA-LUAD dataset, we calculated the *p*-value for common DEGs and selected the significant survival-related DEGs (*p*-value<0.05). In previous studies, the optimal prognostic biomarkers were selected by multivariate Cox proportional hazard models (Chen et al., 2019; Yuan, Ren, & Wang et al., 2021) or stepwise regression with backward selection (Wang et al., 2020). To minimize the possibility of overfitting as much as possible, we used LASSO Cox regression analysis to identify the appropriate genes. LASSO is a compression estimation method that can compress the regression coefficients by constructing a penalty function for selecting variables (Z. Yu et al., 2022). By LASSO, the genes with the non-zero coefficient after the shrinking process were selected to construct the prognostic model. The advantages of LASSO include avoiding overfitting, automatic feature selection, and short processing time. A previous study, containing the comparison of models, showed the performance of LASSO is better than stepwise regression (Kumar et al., 2019). Using the glmnet package (Friedman et al., 2010), the LASSO has been successfully applied for survival prognosis in many application areas, including oncology (Yuan, Ren, & Li et al., 2021; Zhang et al., 2020).

### 2.5 Validation of the signature

Risk scores of TCGA-LUAD samples were generated by the expression of genes and the corresponding coefficient, which was calculated by univariate Cox analysis. Relying on the median value, TCGA-LUAD individuals were then split evenly into high- and low-risk groups. Additionally, we built the risk score to anticipate the prognosis of TCGA-LUAD. In addition, in order to test the accuracy, we constructed calibration curves and calculated the AUC. Calculations were made to determine the degree of connection between risk score and clinicopathological characteristics. Following that, univariate and multivariate Cox regression analyses were carried out with the goal of determining whether or not the risk score was an independent risk factor. An independent dataset (GSE37745) was randomly selected as the testing dataset for validating the risk score model.

### 2.6 Construction and validation of the nomogram

A nomogram was created relying on the risk score and the clinicopathological features, including age, AJCC tumor stage (I, II, III, IV), and gender. The calibration curve was plotted to estimate the model’s effectiveness. The discrimination capacity was then computed using the concordance index (C-index). The C-index runs between 0.5 and 1.0, with 0.5 suggesting a useless model whatsoever and 1.0 suggesting an excellent model.

### 2.7 Construction and validation of the random forest model to distinguish sensitive and resistant EGFR-TKI patients

In order to combine the expression data of discovery datasets (GSE31625 and GSE34228) and three independent datasets (GSE122005, GSE80344, and GSE123066) and remove the batch effect, the following steps were adopted. 1) the expression data profiles of these five datasets were normalized by the method of “min-max normalization” which scaled data in the range (0, 1). 2) The expression data profiles of these five datasets were combined, and then we used “ComBat” function from the “sva” package to remove the batch effect (Leek et al., 2012). The ‘sva’ package and “ComBat” function were used in multiple studies to eliminate the batch effects (Li et al., 2020; Tang et al., 2020; J. Yu et al., 2022). We evaluated the batch effect by the principal component analysis (PCA) before and after using the “sva” package. In the expression data after the batch effect correction, the expression values of eight risk score genes were used in model construction and evaluation.

Firstly, the expression data of samples from discovery datasets (GSE31625 and GSE34228) was used in model construction. This data was then randomly and evenly divided into the training dataset (50%) and the internal testing dataset (50%). The expression data of three independent datasets (GSE122005, GSE80344, and GSE123066) were used as the external testing dataset. Then, based on the eight genes, we constructed a random forest model by the “caret” R package (Kuhn, 2008) to distinguish sensitive and resistant EGFR-TKI patients on the training dataset. The model was trained with 3-fold cross-validation, which is adopted by studies to get the optimal characteristics (Zhang et al., 2020). ROC plots and AUC values were obtained to evaluate the performance of the constructed model in the internal and external testing datasets.

### 2.8 Estimation of tumor microenvironment

ESTIMATE was utilized to estimate the status of immune and stromal cell infiltration in each cancer tissue (Yoshihara et al., 2013). The relative abundance of immune cells in each LUAD patient was determined by converting the expression levels of genes into the fraction of immune cells. This was accomplished using the R package ‘CIBERSORT’ and the deconvolution-based CIBERSORT method (Chen et al., 2018). The link between risk score and immune cells in LUAD patients was studied in the TCGA dataset. The expression levels of immune checkpoint genes were extracted, including PD-L1 (CD274), PD1 (PDCD1), CTLA-4 (CTLA4), TIM3 (HAVCR2), LAG3, and TIGIT. The comparison analysis was conducted on the LUAD patients from the TCGA dataset.

## 3 Results

### 3.1 Detection of differentially expressed genes in EGFR-TKI sensitive and resistant cells

**FIGURE 1:**
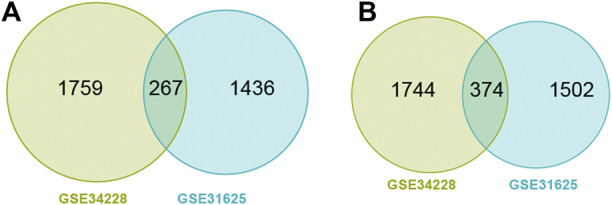
Wayne diagram showing common upregulated DEGs (uDEGs) **(A)** and common downregulated DEGs (dDEGs) **(B)** in the EGFR-TKI resistant LUAD cells.

GSE31625 (erlotinib) and GSE34228 (gefitinib) datasets were selected for detection of EGFR-TKI resistance-related DEGs. A volcano and a heatmap plot from GSE31625 were shown in Supplementary Figure S1A,B. Among 3579 DEGs from GSE31625, there were 1703 highly elevated DEGs and 1876 significantly dDEGs. Similarly, a volcano and a heatmap plot from GSE34228 were shown in Supplementary Figure S2A,B. Among 4144 DEGs from GSE34228, there were 2026 highly elevated DEGs and 2118 significantly dDEGs. In total, 267 shared upregulated genes (Figure 1A) and 374 shared downregulated genes (Figure 1B) were discovered by Wayne analysis of the two datasets.

### 3.2 Function enrichment analysis

These DEGs were then used for GO, KEGG, and Hallmark enrichment analyses, and the top 10 enrichment terms were shown in Supplementary Table S2–6. In the BP (Supplementary Table S2), uDEGs were enriched in cold-induced thermogenesis (GO:0120162), bone resorption (GO:0045780), and neuromuscular junction development (GO:0007528). In the CC (Supplementary Table S3), the uDEGs were mainly enriched in the mitochondrial matrix (GO:0005759), neuromuscular junction (GO:0031594), and mitochondrial inner membrane (GO:0005743). In the MF category (Supplementary Table S4), the uDEGs were enriched in NADPH binding (GO:0070402), oxidoreductase activity (GO:0016628), and ribose phosphate diphosphokinase activity (GO:0004749). In the KEGG analysis (Supplementary Table S5), uDEGs were primarily involved in oxytocin, apelin, and GnRH signaling pathways. In the Hallmark enrichment analysis (Supplementary Table S6), uDEGs were primarily involved in epithelial mesenchymal transition (EMT), fatty acid metabolism, and adipogenesis.

Similarly, in the BP (Supplementary Table S2), dDEGs were enriched in defense responses to symbiont (GO:0140546), defense responses to virus (GO:0051607), and cytokine-mediated signaling pathways (GO:0019221). In the CC (Supplementary Table S3), the dDEGs were mainly enriched in bounding membrane of organelle (GO:0098588), cytoplasmic vesicle membrane (GO:0030659), and cornified envelope (GO:0001533). In the MF (Supplementary Table S4), the dDEGs were enriched in hydrolase activity (GO:0016813), protein-arginine deiminase activity (GO:0004668), and arachidonic acid binding (GO:0050544). In the KEGG (Supplementary Table S5), dDEGs were primarily involved in coronavirus disease, Hepatitis C, and estrogen signaling pathways. In the Hallmark enrichment analysis (Supplementary Table S6), dDEGs were primarily involved in interferon gamma response and interferon alpha response.

### 3.3 Calculation of the risk score

**FIGURE 2:**
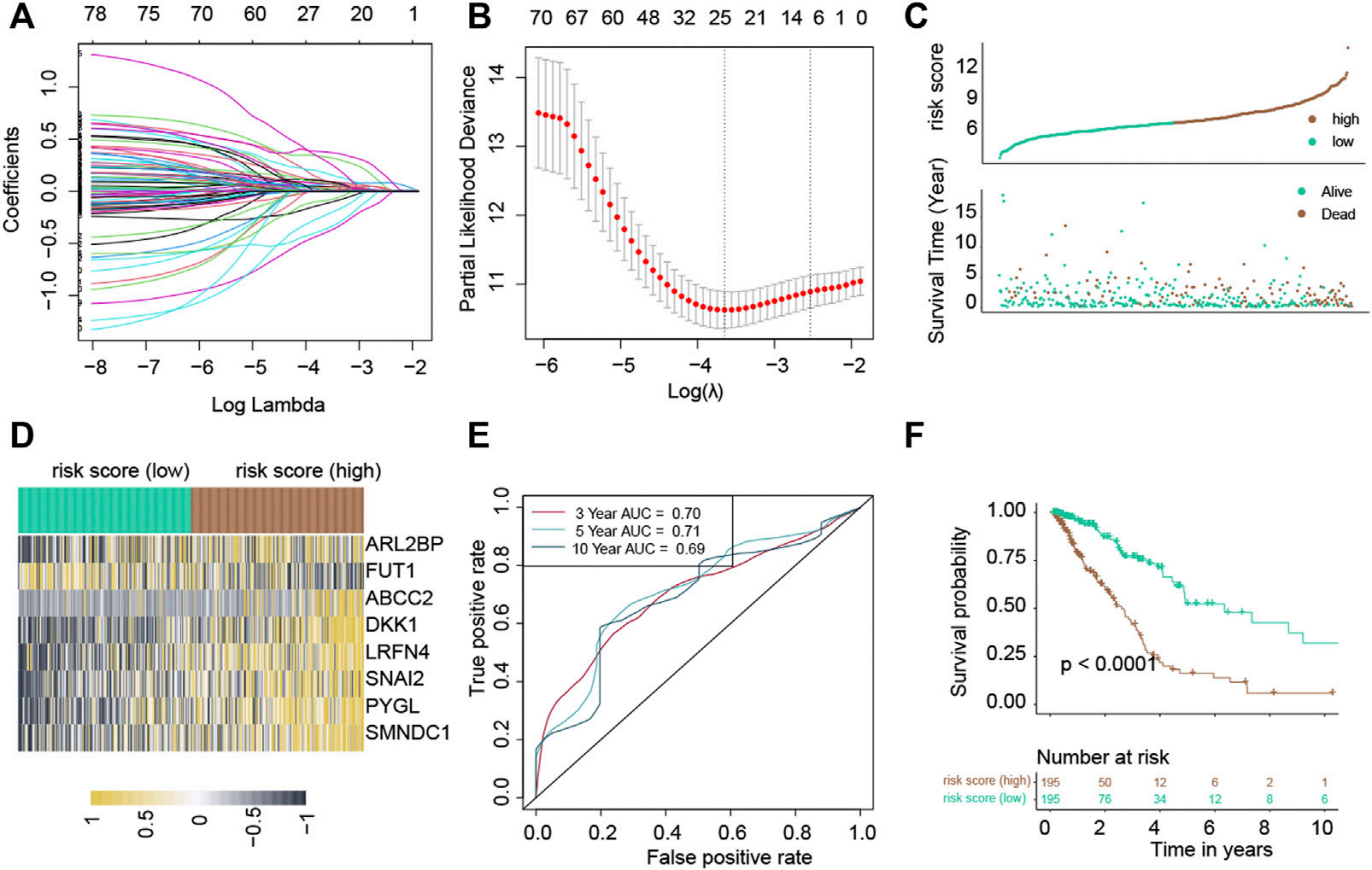
Construction of a risk score in the TCGA-LUAD cohort. The LASSO Cox regression analysis. **(A)** Coefficient values of genes. **(B)** The coefficient plot was plotted against the log(lambda) values. **(C)** Distribution of risk scores and survival summary. **(D)** Heatmap illustrating the expression of the genes in groups with low and high risk. **(E)** The risk score for predicting survival was assessed by AUC values. **(F)** There was a reduced overall survival among individuals in the high-risk group compared to patients in the low-risk group.

Among the 267 common uDEGs, 59 genes with HR>1 and *p*-value<0.05 were defined as risky genes. Similarly, among 374 common dDEGs, 21 genes with HR<1 and *p*-value<0.05 were defined as the protective genes. The LASSO Cox regression algorithm was used to additionally pick these 80 genes in order to create a prognosis signature. Consequently, we identified an 8-gene signature according to the optimal *λ* value (Figures 2A,B). Furthermore, we used the following equation to compute the risk scores of TCGA-LUAD patients: score= (0.16683) * expression_ABCC2_ + (0.6072) * expression _ARL2BP_ + (0.19634) * expression_DKK1_ + (-0.5254) * expression_FUT1_ + (0.36419) * expression_LRFN4_ + (0.35000) * expression_PYGL_ + (0.7995) * expression_SMNDC1_ + (0.34562) * expression_SNAI2_. Following the median risk score, all LUAD individuals were further separated into low-risk and high-risk groups (Figure 2C). In the TCGA-LUAD dataset, the high-risk group experienced higher fatalities (Figure 2C). The mRNA expression of these eight genes in high- and low-risk individuals was compared in Figure 2D. Furthermore, AUC values showed that this risk score predicts prognosis with satisfactory accuracy (3-year: 0.70; 5-year: 0.71; 10-year: 0.69) (Figure 2E). Consistently, the high-risk group of LUAD patients had a lower survival rate than the low-risk (Figure 2F, *p*-value<0.001).

### 3.4 Validation of the risk score

**FIGURE 3:**
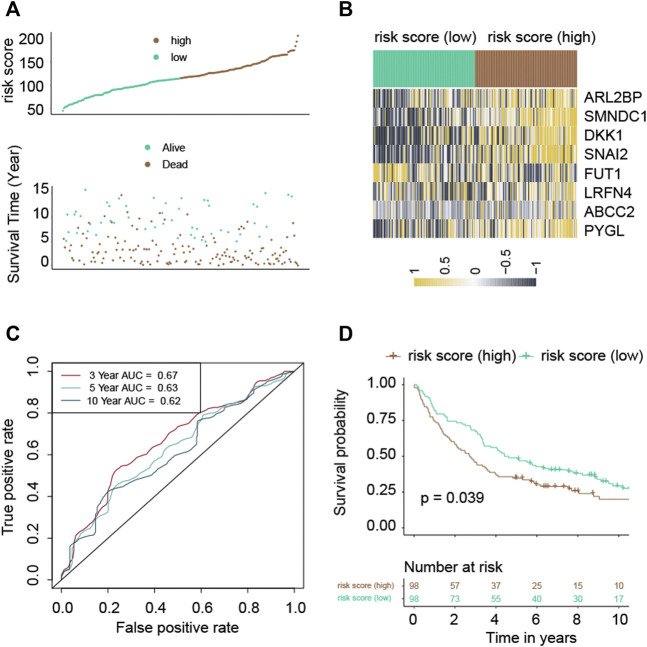
Validation of the risk score in the GSE37745 cohort. **(A)** Distribution of risk scores and survival summary. **(B)** Heatmap illustrating the expression of the genes in groups with low and high risk. **(C)** The risk score for predicting survival was assessed by AUC values. **(D)** There was a reduced overall survival among individuals in the high-risk group compared to patients in the low-risk group.

Following the median risk score, all LUAD individuals from GSE37745 were further separated into low-risk and high-risk groups (Figure 3A). The high-risk group experienced higher fatalities (Figure 3A). The mRNA expression of these eight genes in high- and low-risk individuals was compared in Figure 3B. Furthermore, AUC values showed that this risk score predicts prognosis with satisfactory accuracy (3-year: 0.67; 5-year: 0.63; 10-year: 0.62) (Figure 3C). Consistently, the high-risk group of LUAD patients had a lower survival rate than the low-risk cohort (Figure 3D, *p*-value = 0.039).

### 3.5 Risk score and clinicopathological indicators

Subsequently, the correlation between clinical features and risk score was determined. The risk score did not correlate substantially with the age of LUAD patients (Supplementary Figure S3A). Positive correlations between risk score and AJCC stages (Supplementary Figure S3B), T (Supplementary Figure S3C), N (Supplementary Figure S3D). Male patients were found to be correlated with increased risk score (Supplementary Figure S3F). In contrast, the correlation of the risk score with M (Supplementary Figure S3E) was not significant.

### 3.6 Independent prognostic role of the risk score

**FIGURE 4:**
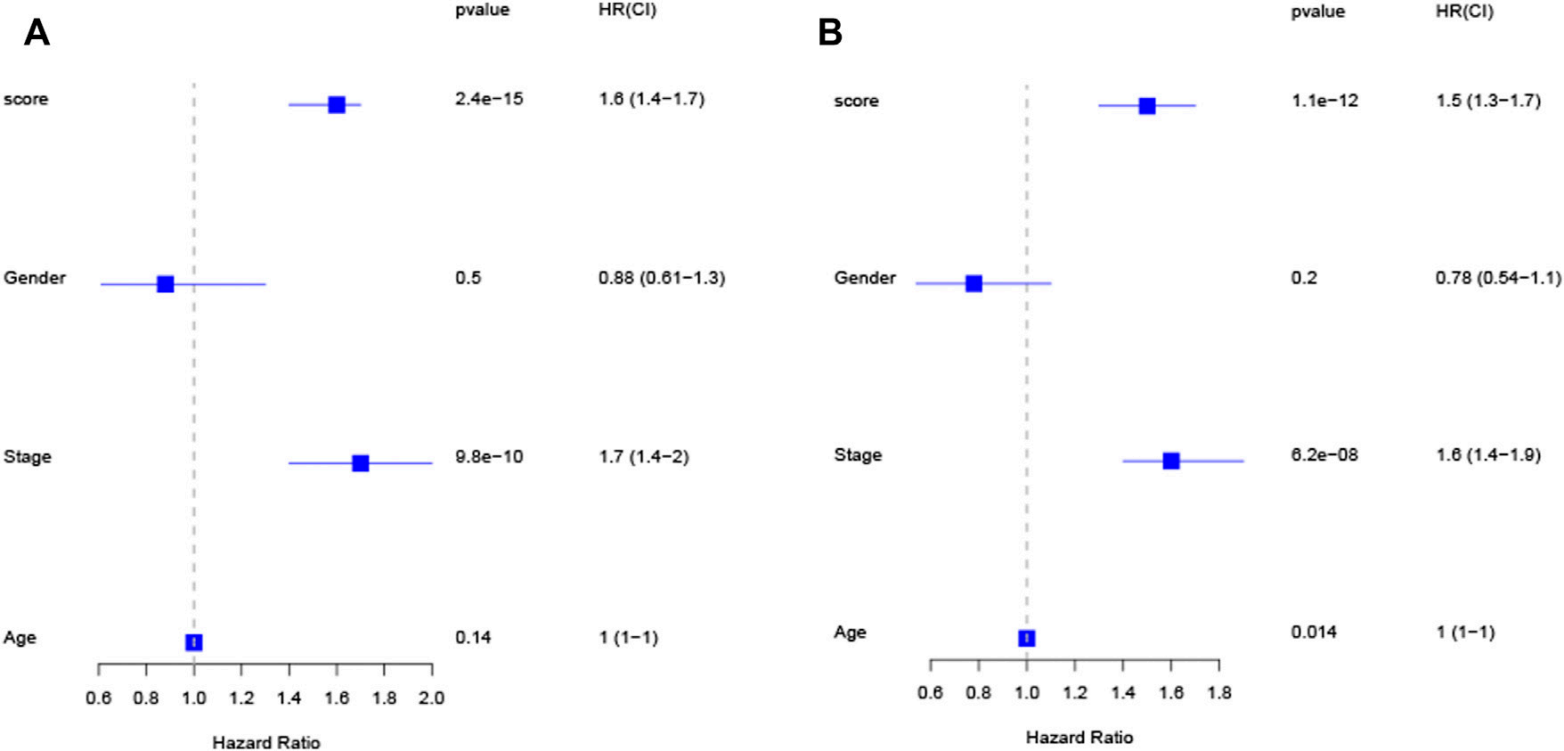
The risk score is an independent predictive factor for LUAD patients in the TCGA cohort, as shown by univariate and multivariate Cox regression analysis **(A,B)**.

Univariate Cox revealed that a higher risk score was strongly connected with poorer survivability (Figure 4A, HR = 1.6, 95% CI: 1.4–1.7) dataset. Similarly, the multivariate Cox indicated that the risk score is an independent predictor of survival when utilizing the TCGA-LUAD (Figure 4B, HR = 1.5, 95% CI: 1.3–1.7) dataset. These findings imply that the risk score has a predictive impact independent of other variables.

### 3.7 Nomogram development and validation

**FIGURE 5:**
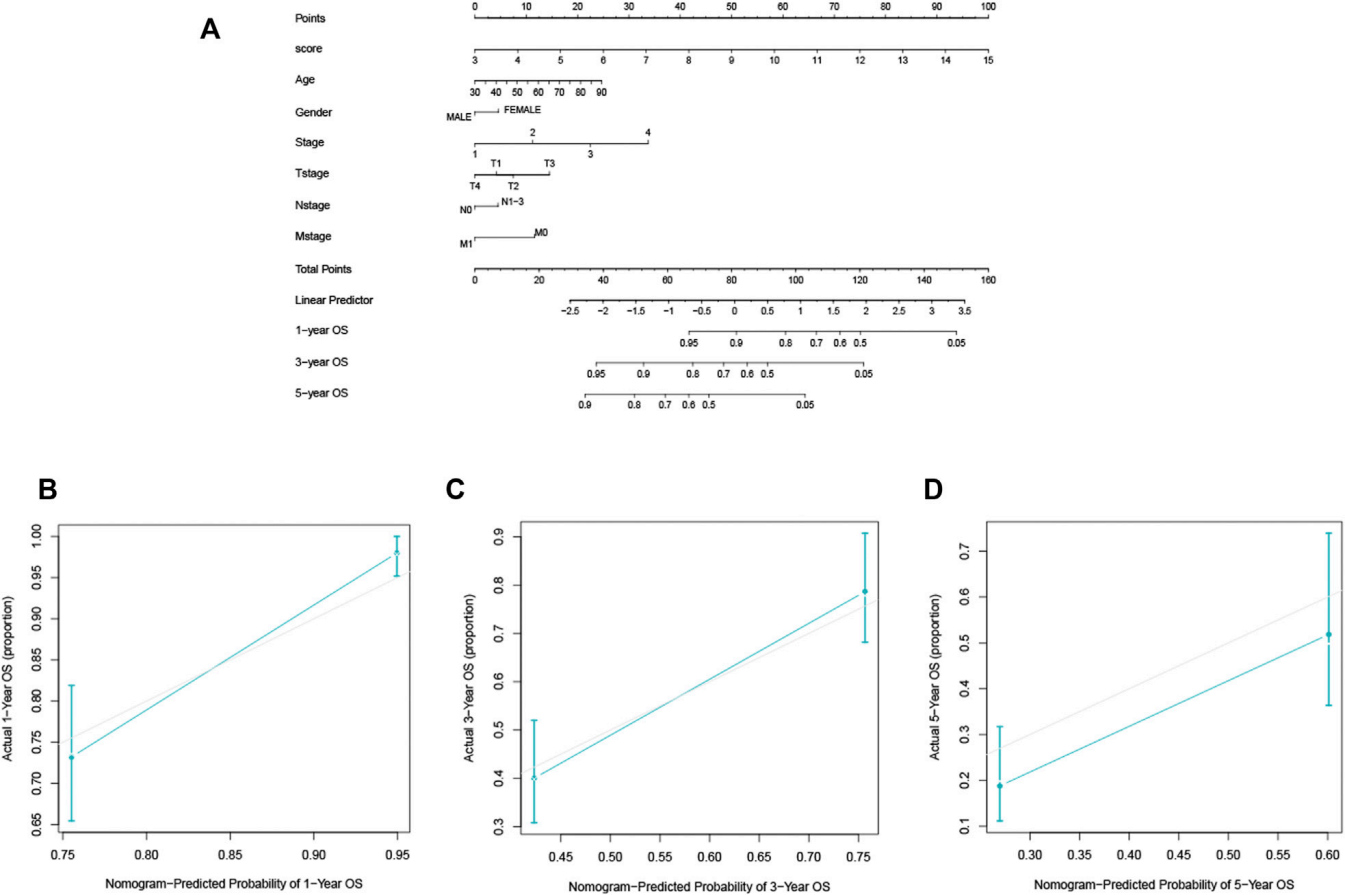
The construction and validation of the nomogram for OS. **(A)** The designed nomogram was used to assess OS. **(B–D)** The calibration curves demonstrated good congruence between the observed likelihood of 1-, 3-, and 5-year survival and the forecast of the nomogram.

In the TCGA-LUAD cohort, 1-, 3-, and 5-year OS were predicted using a nomogram that was constructed by variables: risk score, age, gender, AJCC stages, T, N, and M (Figure 5A). The C-index of the nomogram was 0.77. The calibration plot for the chance of surviving one, three, or five years demonstrated a strong connection between the nomogram’s forecast and actual observation (Figures 5B–D).

### 3.8 Assessing the ability of the risk score to distinguish sensitive and resistant EGFR-TKI samples

As described in the method section, the expression data from the discovery datasets (GSE31625 and GSE34228) and three independent datasets (GSE122005, GSE80344 and GSE123066) were normalized and combined, and then their batch effects were eliminated. Before the batch effect elimination, the heterogeneity of responsive and resistant samples was not found (Supplementary Figure S4A). However, clear batch effects were found among datasets (Supplementary Figure S4B). After the batch effect elimination, responsive samples were separated from resistant samples (Supplementary Figure S4C), and the batch effects among datasets were significantly eliminated (Supplementary Figure S4D). Based on the expression data after the batch effect elimination, training, internal testing, and external testing datasets were defined, respectively.

**FIGURE 6:**
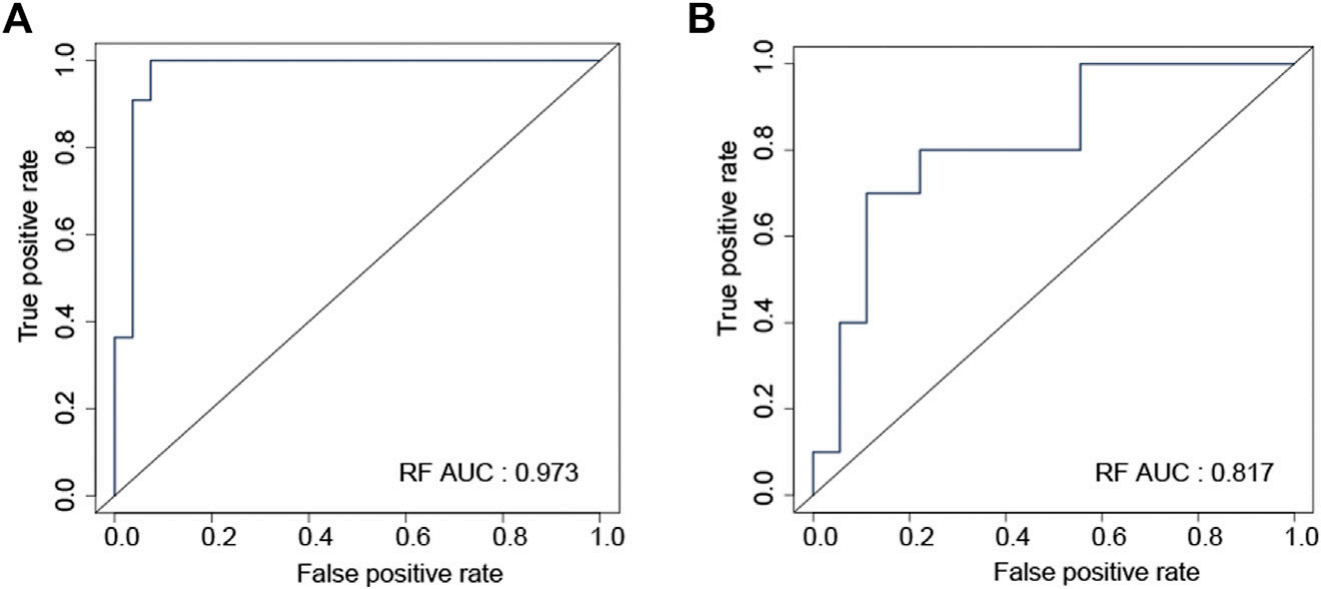
ROC curve validated the sensitivity and specificity of random forest model for distinguishing EGFR-TKI resistant and sensitive samples in the internal testing **(A)** and the external testing **(B)** datasets.

Random forest (RF) was used to construct the prediction model with data from the training dataset, using mRNA expression data of eight genes (ABCC2, ARL2BP, DKK1, FUT1, LRFN4, PYGL, SMNDC1, and SNAI2). In the internal testing dataset, RF model reached an overall predictive AUC of 0.973 (Figure 6A). In the external testing dataset (GSE122005, GSE80344 and GSE123066), the RF model worked wonderfully, with an overall predictive AUC of 0.817 (Figure 6B).

### 3.9 The correlation of risk score with immune status

**FIGURE 7:**
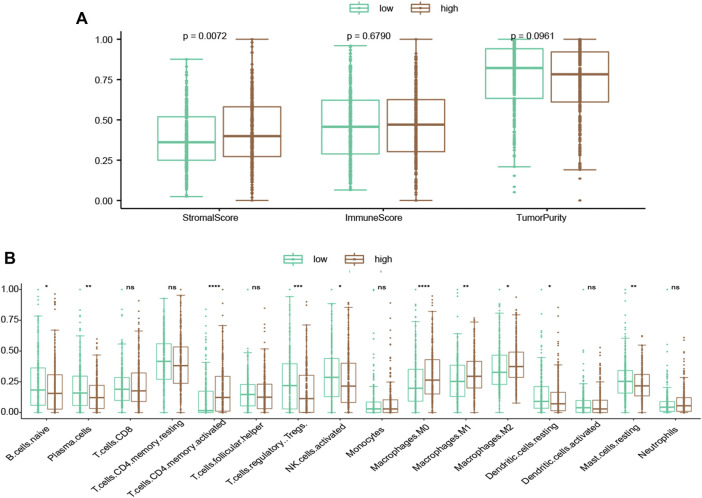
Evaluation of the association of TME compositions with the risk score. **(A)** Inside the high-risk group, the stromal score was greater. **(B)** Inflammatory cells infiltrating ratios in risk groups. */**/****: statistically significant.

In order to assess the immunity status of LUAD patients in the low- and high-risk classes, two distinct techniques were used. According to the ESTIMATE methodology, the stromal score was considerably greater in the high-risk instances (Figure 7A). However, the immune score did not show a significant difference between two groups (Figure 7A; *p* = 0.679). To further investigate the link between risk score and various immune cells, we measured the number of immune cells by CIBERSORT. The low-risk group had considerably more naive B cells, plasma cells, Tregs, activated NK cells, and resting dendritic cells (Figure 7B). On the other hand, more activated CD4 memory cells and macrophages (M0, M1, M2) were present in the high-risk group (Figure 7B). Besides, higher immune checkpoint genes, including PD-L1 (CD274), PD1 (PDCD1), CTLA-4 (CTLA4), TIM3 (HAVCR2), LAG3, and TIGIT were present in the high-risk group (Supplementary Figure S5).

### 3.10 Survival analysis of selected genes

**FIGURE 8:**
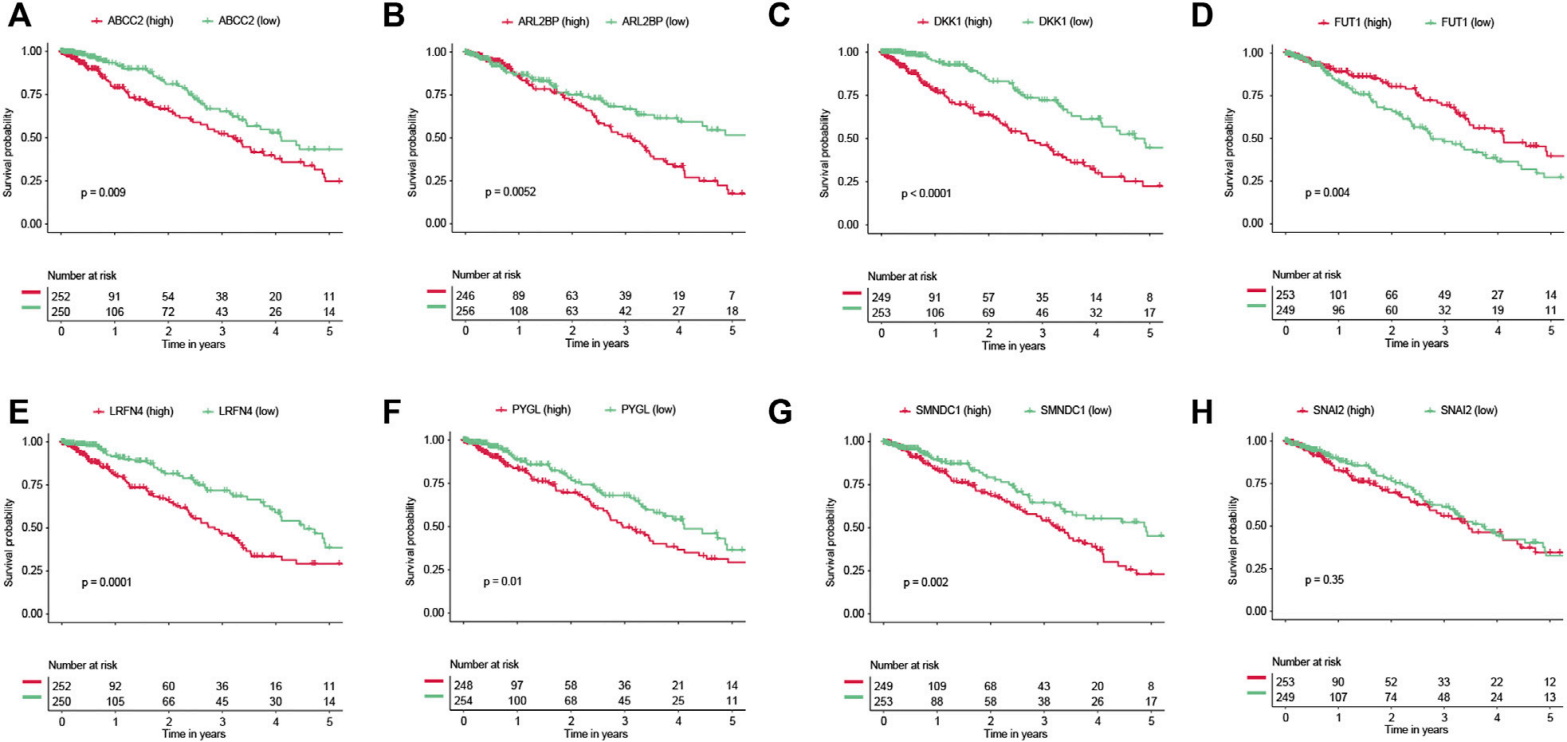
Survival analyses of genes. Prognostic values of **(A)** ABCC2, **(B)** ARL2BP, **(C)** DKK1, **(D)** FUT1, **(E)** LRFN4, **(F)** PYGL, **(G)** SMNDC1, and **(H)** SNAI2.

Six of the eight genes (ABCC2, ARL2BP, DKK1, LRFN4, PYGL, and SMNDC1) were related to worse overall survival (Figures 8A–H). Conversely, FUT1 was related to improved overall survival (Figure 8D). We also evaluated the expression values of these genes between two EGFR-TKI groups. In GSE31625, five (ABCC2, ARL2BP, PYGL, SMNDC1, SNAI2) of eight genes were significantly higher in the EGFR-TKI resistant group (Supplementary Figure S6A). In GSE34228, seven (ABCC2, ARL2BP, DKK1, LRFN4, PYGL, SMNDC1, SNAI2) of eight genes were significantly higher in the EGFR-TKI resistant group (Supplementary Figure S6B). It should be noted that FUT1 was significantly higher in the EGFR-TKI sensitive group by expression from GSE31625 and GSE34228.

## 4 Discussion

LUAD is the most prevalent subtype of NSCLC, accounting for nearly fifty percent of lung malignancies (Jordan et al., 2017). Current therapy for LUAD consists of surgery and pharmaceutical drugs. Currently, three generations of EGFR-TKI have been approved for use. These three generations of EGFR-TKI include erlotinib/gefitinib (first generation), afatinib/dacomitinib (second generation), and osimertinib (third generation). Compared with standard chemotherapy, the EGFR-TKI significantly improved clinical outcomes (Del et al., 2019). Most patients treated with EGFR-TKI will acquire resistance, which significantly restricts the clinical use of EGFR-TKI.

The main purposes of this study include: 1) identifying potential EGFR-TKI resistance-related biomarkers; 2) providing models to distinguish sensitive from resistant EGFR-TKI samples; 3) providing models to predict the prognosis of LUAD. Eight genes (ABCC2, ARL2BP, DKK1, FUT1, LRFN4, PYGL, SMNDC1, and SNAI2) were identified by bioinformatics analysis. We constructed a RF model by these genes to distinguish sensitive and resistant EGFR-TKI samples, and the model reached predictive AUCs of 0.973 and 0.817 in the internal and external testing dataset. We also constructed a risk score model by these genes to predict the LUAD prognosis, and it performed with satisfactory accuracy with an AUC of 0.67 on 3-year prognosis prediction in the independent dataset (GSE37745). Recently, constructing prognostic models by mRNA expression data has become prevalent in many cancer studies. Using 16 metabolic genes, a previous study constructed a prognostic model for LUAD and it reached an AUC value of 0.638 on 3-year prognosis prediction in the independent dataset (GSE37745) (He et al., 2020). Another study provided a risk score model for LUAD by six genes, and it reached an AUC value of 0.66 on 3-year prognosis prediction in GSE37745 (Jiang et al., 2022). In a LUAD prognosis model constructed by 27 hypoxia-related genes, it showed AUC values of 0.65 and 0.66 in the validation datasets (Ouyang et al., 2021). Together, these findings imply that our risk model is more accurate, stable, and capable of accurately reflecting the prognosis of LUAD patients.

The data in GSE31625 and GSE34228 datasets were extracted to compare gene expression between EGFR-TKI sensitive and resistant cell samples, and the common DEGs were screened out. Enrichment analysis indicated that the majority of commonly up-regulated DEGs were enriched in pathways associated with EMT, fatty acid metabolism, and adipogenesis. During EMT, epithelial cells transform into mesenchymal cells. Studies have been developed to show that EMT is engaged in the metastasis, related with the growth of many different types of malignancies, and connected with chemoresistance (Xiao et al., 2010), such as resistance to EGFR-TKI (Clement et al., 2020; Thomson et al., 2005). There is a correlation between the expression of mesenchymal markers and a poor prognosis as well as a suboptimal response to EGFR-TKIs in NSCLC. This is because mesenchymal markers contribute to a resistant phenotype (Jakobsen et al., 2016).

According to the findings of this investigation, the levels of expression of seven genes—ABCC2, ARL2BP, DKK1, LRFN4, PYGL, SMNDC1, and SNAI2—were higher in resistant samples than in sensitive ones. Previous research indicated that EGFR-TKI-resistant cells had higher levels of ABCC2 expression. (Hamamoto et al., 2017). One of the studied inhibitors of canonical Wnt signaling is a protein called DKK1 (Chu et al., 2021). DKK1 is found to be substantially more expressed in lung cancer tissues than normal controls. In addition, a number of recent studies have shown that DKK1 is positively correlated with lung cancer stage and tumor metastasis, and that it may promote lung cancer invasion and proliferation (Song et al., 2019).

The limitations of the present study should be mentioned. 1) The available public expression datasets are quite limited. We have combined the gene expression data from three independent datasets to validate the performance of the model on distinguishing EGFR-TKI resistant and sensitive samples. However, the sample size is still limited and a new cohort with more samples should be used to validate the model. 2) These eight genes have a high association with EGFR-TKI resistance in different datasets. However, the mechanism of these eight genes affecting EGFR-TKI resistance should be investigated by further experiments.

## 5 Conclusion

In conclusion, the eight genes linked to EGFR-TKI resistance were significantly connected with the prognosis of LUAD. The machine learning model based on these eight genes showed high accuracy in distinguishing EGFR-TKI resistant and sensitive samples. The risk score and the nomogram based on these eight genes showed high accuracy in predicting the survival outcome. Through our research, we were able to find eight genes that are linked to EGFR-TKI resistance and provide models that can predict EGFR-TKI resistance and the prognosis for LUAD patients(Yu and Ouyang, 2022).

## Data Availability

The datasets presented in this study can be found in online repositories. The names of the repository/repositories and accession number(s) can be found in the article/Supplementary Material.
